# Reversed Dipper Blood-Pressure Pattern Is Closely Related to Severe Renal and Cardiovascular Damage in Patients with Chronic Kidney Disease

**DOI:** 10.1371/journal.pone.0055419

**Published:** 2013-02-05

**Authors:** Cheng Wang, Jun Zhang, Xun Liu, Cuicui Li, Zengchun Ye, Hui Peng, Zhujiang Chen, Tanqi Lou

**Affiliations:** Division of Nephrology, Third Affiliated Hospital of Sun Yat-Sen University, Guangzhou, Guangdong, China; Rouen University Hospital, France

## Abstract

**Background:**

A non-dipper blood pressure (BP) pattern is very common in chronic kidney disease (CKD) patients and affects the progression and development of cardiovascular disease. However, data on the reversed dipper BP pattern on target-organ damage in Chinese CKD patients are lacking.

**Methods:**

A total of 540 CKD patients were enrolled. Ambulatory blood pressure monitoring (ABPM), clinical BP, ultrasonographic assessment and other clinical data were collected. Univariate and multivariate analyses were used to ascertain the relationship between ABPM results and clinical parameters.

**Results:**

A total of 21.9% CKD patients had a reversed dipper BP pattern, 42% of patients had a non-dipper BP pattern and 36.1% of patients had a dipper BP pattern. Patients with reversed dipper BP pattern had the worst renal function and most severe cardiovascular damages among these CKD patients (p<0.05). The estimated glomerular filtration rate (eGFR) and left ventricular mass index (LVMI) correlated significantly with the rate of decline of nocturnal BP. A reversed dipper BP pattern was an independent factor affecting kidney damage and left ventricular hypertrophy. Age, lower hemoglobin level, higher 24-h systolic BP from ABPM, and higher serum phosphate levels were independent associated with a reversed dipper BP pattern after multivariate logistic regression analyses.

**Conclusion:**

The reversed dipper BP pattern is closely related to severe renal damage and cardiovascular injuries in CKD patients, and special attention should be given to these CKD patients.

## Introduction

The rising prevalence and associated morbidity of chronic kidney disease (CKD) has resulted in significant disease burden and became a major public health problem in many countries [1]. Hypertension is the most prevalent independent risk factor for the progression of renal failure and cardiovascular disease [2]. Antihypertensive agents reduce blood pressure (BP), lower urinary excretion of protein and delay the progression of kidney disease [3]. The sixth report of the Joint National Committee for the Prevention, Detection, Evaluation, and Treatment of High Blood Pressure (JNCVI) recommends a lower BP goal for patients with reduced kidney function (<130/85 mmHg if urinary protein excretion is <1 g/d and <125/75 mmHg if it is >1 g/d) than for patients without target-organ damage (<140/90 mmHg) [4].

Accurate measurement of BP is very important for CKD patients, whereas the traditional Perloff method has limitations (it can provide only an incomplete and potentially misleading assessment of the severity of hypertension) [5]. Ambulatory blood pressure monitoring (ABPM) is available not only in specialized clinics but also in many segments of primary care. Several studies have demonstrated the better reproducibility and prognostic superiority of BP values obtained using ABPM as compared with BP values obtained from standard clinical measurements [6]. The prognostic value of ABPM has been established for the general population as well as for hypertension in general or under specific conditions (e.g., refractory hypertension, diabetes mellitus). Clinical observations have identified subjects with a blunted decline in the nocturnal fall of physiological BP: the “non-dipper” pattern, while subject whose nocturnal BP reduction was “normal” (defined as a drop of >10% of the diurnal BP value [7]). Subjects with a non-dipper pattern have more target-organ damage induced by hypertension such as left ventricular hypertrophy, microalbuminuria, and reduced arterial compliance. Furthermore, follow-up studies have demonstrated that non-dipper subjects have a worse prognosis in terms of cardiovascular events as well as a higher risk of developing diabetic nephropathy in normotensive type-1 diabetic mellitus [8], [9]. However, non-dipper patients in these studies were mixed groups comprising individuals with reversed and non-dipper BP patterns. This begs several questions: what is the frequency of reversed dipper BP pattern in CKD patients residing in China? Do these subjects with reversed BP have worse renal and cardiovascular damage compared with those with non-dipper BP patterns? In theory, patients with reversed dipper BP should have severe cardiovascular and renal damages, while there are no data on Chinese CKD patients with reversed dipper BP pattern. Answering these two questions in Chinese populations is very important considering the different environment, life style and primary causes of CKD compared with western countries. Hence, we carried out an observational study to identify the features and frequency of reversed dipper BP in CKD patients residing in China.

## Materials and Methods

### Study Design

All patients in our division diagnosed with CKD from May 2010 to May 2012 were enrolled this study. The exclusion criteria were subjects who: (i) had undergone treatment with corticosteroids or hormones; (ii) had acute changes in the estimated glomerular filtration rate (eGFR) >30% in the previous 3 months; (iii) were pregnant; (iv) had a history of abuse of drugs or alcohol; (v) had night-work or shift-work employment; (vi) had acquired immunodeficiency syndrome (AIDS); (vii) had cardiovascular disorders (unstable angina pectoris, heart failure, life-threatening arrhythmia, atrial fibrillation, kidney failure, or grade III–IV retinopathy); (viii) could not tolerate ABPM; (ix) could not communicate and comply with all of the study requirements.

The study protocol was approved by the Ethics Committee of the third hospital of Sun Yat-sen University (Guangzhou, China). All study participants provided written informed consent before enrolling in the study.

### Diagnostic Criteria

CKD patients were diagnosed using clinical practice guidelines set by National Kidney Foundation Disease Outcomes Quality Initiative (NKF-K/DOQI) [10]. The diagnosis of hypertension was based on accepted ABPM criteria [5]. Ambulatory blood pressure (ABP) was considered to be normal if the mean awake BP was <135/85 mmHg for systolic blood pressure (SBP)/diastolic blood pressure (DBP) or if the mean bedtime BP was <120/70 mmHg. “Dipper” was defined as a decline in the nocturnal BP of >10%, whereas “non-dipper” was defined as a decline in the nocturnal BP of <10% but >0%, and “reversed dipper” was defined as a decline in the nocturnal BP of <0%.

#### ABPM

Patients underwent 24-h ABPM using a TM-2430 monitor (A&D Company, Tokyo, Japan). Cuff size was chosen on the basis of arm circumference, and the cuff fixed to the non-dominant arm. Three BP readings were obtained in the morning (7∶00 to 10∶00 am) concomitant with sphygmomanometric measurements to ensure that the mean of the two sets of values differed by <5 mmHg. BP was recorded every 15 min from 7∶00 am to 10∶00 pm, and every 30 min from 10∶00 pm to 7∶00 am. The daytime and night-time periods were derived from diaries recorded by the patients during ABPM. Monitoring was always undertaken on a working day. Patients did not have access to values of ABP. Strenuous physical activity was discouraged in all patients during the monitoring period. Their daily activities were comparable. BP series were eliminated from the analysis if: >30% of the measurements were missing; data were missing for >3-h spans; they were collected from subjects while they were experiencing an irregular rest–activity schedule or a night-time sleep span <6 h or >12 h during monitoring.

### BP Measurement in the Physician’s Office

During a visit to the physician (8∶00 to 11∶00 am), BP was measured for each patient [11]. Briefly, measurements were taken in a quiet environment using a mercury sphygmomanometer with the patient in a sitting position after 5 minute of rest. BP was not measured if the patient had consumed tobacco, ingested caffeine, or eaten within 30 min. SBP and DBP values (Korotkoff’s phase I and phase V, respectively) at each visit enabled recording of a minimum of two BP measurements at intervals of ≥1 min. For each visit (baseline and after the drug shift), the reported values of office BP were the mean of values recorded during the 2 days in which the ABP device was installed and removed. For all patients, sphygmomanometric measurements were recorded by the same physician, who was not aware of the results of ABP recordings.

### Ultrasonographic Assessment

The volume, mass, systolic function and diastolic function of the left ventricle were assessed using two-dimensional echocardiography. Left ventricular mass was calculated using the Duvereux method [12]. The left ventricular mass index (LVMI) was obtained by calculating left ventricular mass to height^2.7^ [13], and patients with LVMI >49 g/m^2.7^ (man) and >45 g/m^2.7^ (woman) were diagnosed as left ventricular hypertrophy (LVH) [14]. Left ventricular systolic function was assessed by left ventricular ejection fraction (LVEF). Diastolic function was assessed by early mitral inflow filling velocity (E), peak mitral filling velocity at atrial contraction (A), E/A deceleration time of the mitral E wave, and the tissue Doppler velocity of the mitral annulus [15].

Carotid intima-media thickness (CIMT) was determined by averaging three measurements taken on each carotid artery (in anterior, lateral and posterior directions), measuring the distance between the leading edge of the lumen–intima interface, and the leading edge of the collagenous upper layer of the adventitia using high-resolution B mode ultrasonography. Measurements were taken in areas free of obvious atherosclerotic plaques around the level of the carotid bifurcation.

### Other Data Collected

We collected 24-h urine samples within a 7-day period of ABPM and measured levels of urinary protein, creatinine, and sodium excretion. We also collected urine samples at 7∶00 am to 10∶00 pm and 10∶00 pm to 7∶00 am to detect proteinuria and sodium excretion (which were termed “awakening and bedtime urine proteinuria” and “awakening and bedtime sodium excretion, respectively”). Patients were asked to void their bladders at 7∶00 am and 10∶00 pm to ensure valid results. In addition, medical history, including demographic and laboratory data (serum levels of creatinine, cholesterol, triglycerides (TGs), high-density lipoprotein-cholesterol (HDL-C), low-density lipoprotein-cholesterol (LDL-C), fasting glucose), and current therapy were obtained at the initial study visit.

### Statistical Analyses

Data were analyzed using SPSS ver15.0 (SPSS, Chicago, IL, USA). Descriptive statistics are presented as percentages for discrete variables and as means (standard deviation) for continuous variables. Non-parametric variables are expressed as median and interquartile range. Comparisons for continuous variables between groups were tested by the Student’s *t* test, analysis of variance (ANOVA), or non-parametric test. The relationship between two continuous variables was assessed by a bivariate correlation method (Pearson’s correlation).

Logistic regression analyses were used to explore the associations of three types of CKD outcomes: eGFR (eGFR<60 mL/min/1.73 m^2^ *vs* eGFR ≥60 mLl/min/1.73 m^2^), 24-h proteinuria (≥1 g/24 h *vs* <1 g/24 h) and LVH (no LVH *vs* LVH) and reversed dipper status. CKD outcomes were treated as dependent variables. Sex, age, clinical variables, all BP values from the clinic BP and ABPM, and dipping status (1 = non-reversed dipper, 2 = reversed dipper) were treated as independent variables. Logistic regression analyses were also used to identify the factors associated with dipper status (non-reversed dipper *vs* reversed dipper) (dependent variables) and age, sex, clinical variables, clinic- SBP and DBP and 24-h SBP and DBP (independent variables). To explore the potential confounding factors, all those variables mentioned above which significantly correlated with dependent variables were tested further in multiple logistic regression models with the forward likelihood method. Univariate linear regression model was employed to examine the association between E/A ratio (dependent variable) and age, sex, eGFR(1 = eGFR≥60 mL/min/1.73 m^2^; 2 = eGFR<60 mL/min/1.73 m^2^), levels of hemoglobin, serum calcium, phosphate and intact parathyroid hormone (iPTH), clinic BP and ABPM, and dipper status (1 = non-reversed dipper, 2 = reversed dipper) (independent variables). Those variables with significant associations with the E/A ratio were tested further in multiple linear regression analyses with the forward likelihood method. All values are two-tailed, and significance was defined as p<0.05.

## Results

### 1. Prevalence of a Reversed Dipper BP Pattern among CKD Patients

A total of 118 patients (21.9%) had a reversed dipper BP pattern. A total of 227 patients (42%) had a non-dipper BP pattern, and 195 patients (36.1%) had a dipper BP pattern. The prevalence of the reversed dipper BP pattern in CKD stage I, II, III, IV and V was 12.3%, 8.6%, 18.8%, 36.1% and 35.7% respectively. The prevalence of the reversed dipper BP pattern in patients with an eGFR <60 mL/min/1.73 m^2^ (patients in CKD stages III, IV and V) was higher than that in patients with an eGFR ≥60 mL/min/1.73 m^2^ (patients in CKD stages I and II) (31.3% *vs* 11.1% p<0.05).

### 2. Patients with Reversed Dipper BP Pattern had the Worst Renal and Cardiovascular Damage among CKD Patients

Compared with patients with dipper BP pattern, subjects with reversed dipper BP pattern were older and had: higher clinical SBP, 24-h-SBP, awakening SBP and bedtime BP; a higher ratio of bedtime/awakening time of proteinuria and urinary sodium excretion; lower eGFR; lower hemoglobin, higher serum phosphate, iPTH and uric acid; higher LVMI, lower E/A ratio, higher CIMT and percentage of atherosclerotic plaques around the level of the carotid bifurcation (p<0.05).

More importantly, when we compared patients with reverse dipper BP pattern with patients with a non-dipper BP pattern, we found that patients with reversed dipper BP pattern also had: higher clinical SBP, 24-h SBP, awakening SBP and bedtime BP; higher ratio of bedtime/awakening time of proteinuria and urinary sodium excretion; lower eGFR; lower hemoglobin level, higher serum phosphate and iPTH; higher LVMI and lower E/A ratio (p<0.05). Patients with a reversed dipper BP pattern have the lowest eGFR, highest LVMI and lowest E/A ratio among these CKD patients (Table 1).

**Table 1:** Differences in demographic and clinical features in Chinese CKD patients with dipper, non-dipper, and reversed dipper BP patterns.

	Dipper group	Non-dipper group	Reversed dipper group
	(n = 195)	(n = 227)	(n = 118)
Age(years)	39±16	41±16	50±15* #
Male:female ratio	125∶70	138∶89	59∶59
Duration(months)	24±15	24±15	28±23
BMI(kg/m^2^)	23±4	23±5	23±4
Number of diabetic mellitus	8∶195	4∶227	17∶118* #
Clinic- SBP(mmHg)	137±22	141±25	149±25* #
Clinic- DBP(mmHg)	84±12	85±16	86±14
24 h-SBP from ABPM (mmHg)	129±20	134±19*	143±19* #
24 h-DBP from ABPM (mmHg)	77±11	78±12	82±11
SBP-awakening(mmHg)	132±19	134±19	141±18* #
DBP-awakening(mmHg)	80±11	81±12	82±10
SBP-bedtime(mmHg)	110±30	125±27*	143±32* #
DBP-bedtime(mmHg)	66±19	75±16*	82±18* #
Proteinuria(g/24 h)	1.2(0.4–2.9)	1.4(0.5–3.2)	1.3(0.6–3.7)
Ratio of bedtime/awakening time of proteinuria	0.57±0.42	0.63±0.62	0.85±0.75* #
Urinary sodium excretion(mmol)	130±87	133±69	123±56
Ratio of bedtime/awakening time of urinary sodium excertion	0.5(0.3–0.7)	0.6(0.3–1.0)	0.9(0.5–1.5)* #
Hemoglobin(g/L)	124±26	114±30*	94±27* #
Serum Cystatin C(mg/L)	1.2(0.9–2.5)	1.57(1.1–3.2)	3.8(1.6–5.5)* #
eGFR(mL/min/1.73 m^2^)	76(27–111)	58(16–95)	16(6–48)* #
Cholesterol(mmol/L)	6.3±3.2	6.1±3.2	5.4±2.9* ^#^
Triglyceride(mmol/L)	1.8(1.3–2.7)	1.7(1.1–2.8)	1.7(1.1–2.5)
LDL-C(mmol/L)	4.2±2.6	3.9±2.3	3.3±2.1* #
HDL-C(mmol/L)	1.3±0.5	1.2±0.5	1.1±0.5
Glucose(mmol/L)	4.9±1.3	5.3±2.1	5.6±2.5*
Calcium(mg/dL)	8.8±0.9	8.6±1.0	8.3±1.0*
Phosphate(mmol/L)	4.1±1.2	4.4±1.5	5.2±2.0* #
iPTH(pg/ml)	106±73	157±132*	250±196* #
Uric acid(mmol/L)	429±132	468±165*	482±160*
Homocysteine (µmol/L)	15±8	17±9*	18±9*
LVEF (%)	65±15	63±17	63±17
LVMI(g/m^2.7^)	45±16	52±24*	58±19* #
E/A ratio	1.2±0.5	1.2±0.4	1.0±0.4* #
CIMT(mm)	0.6±0.2	0.7±0.3*	0.7±0.3*
plaque	22/195	34/345*	23/345*

(Serum calcium is corrected by the following formula:[serum calcium] (mg/dL) = measured [serum calcium] (mg/dL)+(4.0−[serum albumin(mg/dl)]) ×0.8. ABPM: ambulatory blood pressure monitoring; BMI: body mass index; CIMT: carotid intima-media thickness; DBP: diastolic blood pressure; eGFR: estimated glomerular filtration rate; E: early mitral inflow filling velocity, A: peak mitral filling velocity at atrial contraction; HDL-C: high-density lipoprotein cholesterol; iPTH: intact parathyroid hormone; LDL-C: low-density lipoprotein cholesterol; LVEF: left ventricular ejection fraction; LVMI: left ventricular mass index LVMI; SBP: systolic blood pressure.

* indicated control with dipper group p<0.05,

# indicated control with non-dipper group p<0.05).

**Figure 1:**
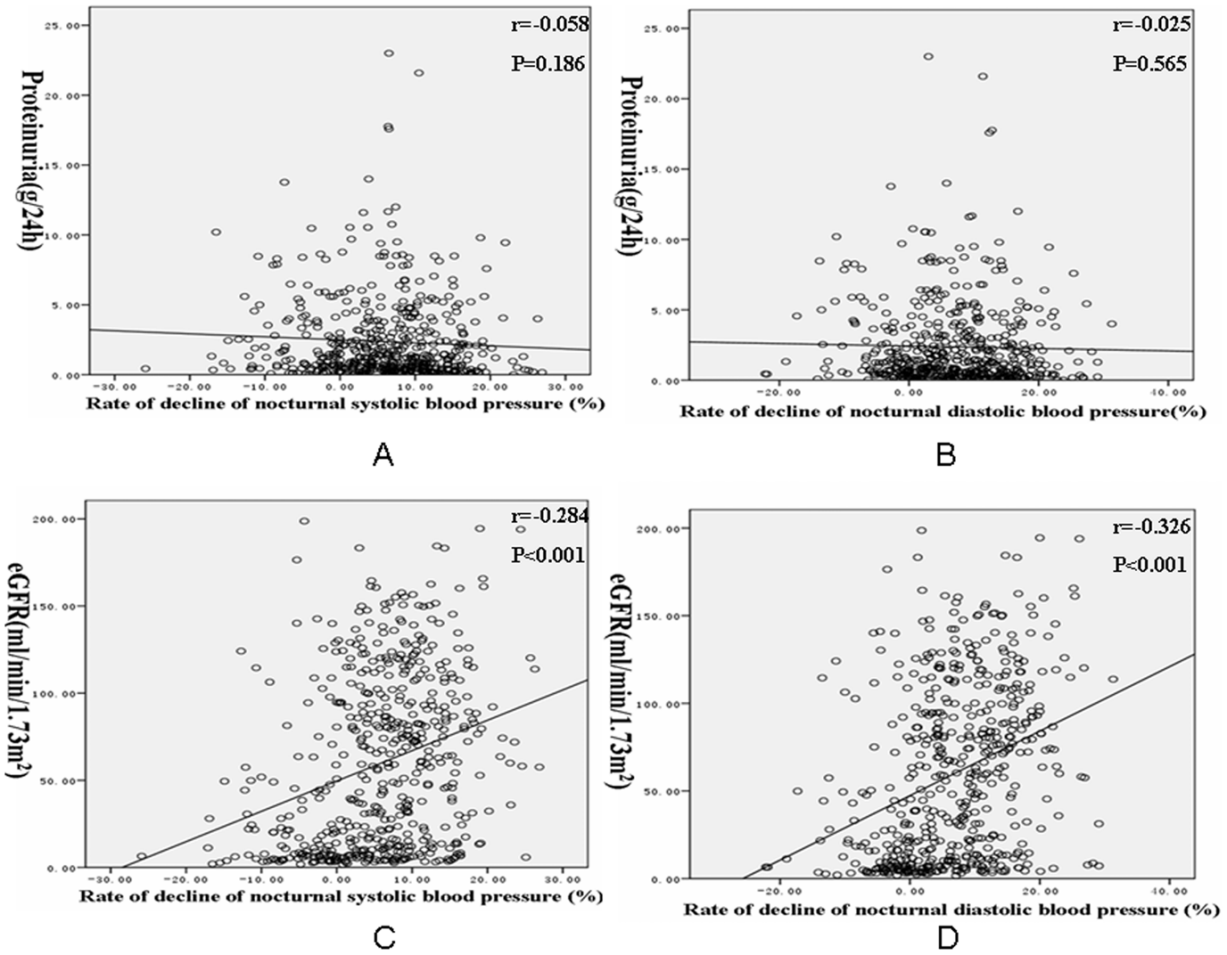
Correlation of proteinuria and estimated glomerular filtration rate with the rate of decline of nocturnal blood pressure. A: Correlation of proteinuria with the rate of decline of nocturnal systolic blood pressure. B: Correlation of proteinuria with the rate of decline of nocturnal diastolic blood pressure. C: Correlation of estimated glomerular filtration rate with the rate of decline of nocturnal systolic blood pressure. D: Correlation of estimated glomerular filtration rate with the rate of decline of nocturnal diastolic blood pressure (eGFR: estimated glomerular filtration rate).

**Figure 2:**
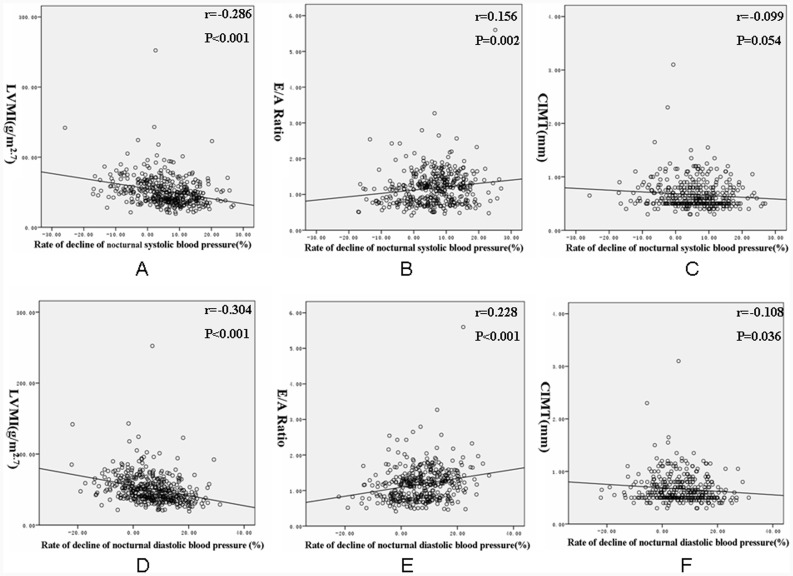
Correlation of left ventricular mass index, E/A ratio and CIMT with the rate of decline of nocturnal blood pressure. A: Correlation of left ventricular mass index with the rate of decline of nocturnal systolic blood pressure. B: Correlation of E/A ratio with the rate of decline of nocturnal systolic blood pressure. C: Correlation of CIMT with the rate of decline of nocturnal systolic blood pressure. D: Correlation of left ventricular mass index with the rate of decline of nocturnal diastolic blood pressure. E: Correlation of E/A ratio with the rate of decline of nocturnal diastolic blood pressure. F: Correlation of CIMT with the rate of decline of nocturnal diastolic blood pressure. (LVMI: left ventricular mass index LVMI; E :.early mitral inflow filling velocity, A: peak mitral filling velocity at atrial contraction; CIMT: carotid intima-media thickness).

Bivariate correlation analysis revealed that eGFR was positively correlated with the rate of decline of nocturnal SBP and DBP, whereas proteinuria of 24 h was not significantly related to the rate of decline of nocturnal SBP and DBP. LVMI was negatively related to the rate of decline of nocturnal SBP and DBP. The E/A ratio was positively correlated with the rate of decline of nocturnal SBP and DBP. CIMT only negatively correlated with rate of decline of nocturnal DBP (Fig. 1 and 2).

### 3. The eGFR in these CKD Patients was Correlated with a Reversed Dipper BP Pattern

Age, levels of hemoglobin, serum phosphate and iPTH, all BP values from the clinic and ABPM, and reversed BP patterns were significantly correlated with the eGFR according to univariate analyses. Multivariate logistic regression analyses revealed that older age, lower hemoglobin, higher serum iPTH level, higher 24-h DBP from ABPM and reversed dipper SBP independently correlated with eGFR<60****mL/min/1.73 m^2^ (Table 2).

**Table 2:** Univariate and multivariate logistic regression analysis for eGFR(1 = eGFR≥60 mL/min/1.73 m^2^; 2 = eGFR<60 mL/min/1.73 m^2^).

	Univariate regression analysis		Multivariate regression analysis	
	OR(95% CI)	P value	OR(95% CI)	P value
Age(per 1 y)	1.065(1.051–1.080)	<0.001	1.046(1.027–1.065)	<0.001
Sex(1 = male, 2 = female)	0.811(0.575–1.145)	0.234		
Hemoglobin(per 1 g/L)	0.967(0.959–0.975)	<0.001	0.962(0.950–0.975)	<0.001
Calcium(per 1 mg/dl)	0.749(0.368–1.527)	0.427		
Phosphate(per 1 mgl/dL)	9.227(5.136–16.579)	<0.001		
iPTH(per 1 pg/ml)	1.024(1.018–1.030)	<0.001	1.014(1.008–1.021)	<0.001
SBP-awakening(per 1 mmHg)	1.065(1.051–1.078)	<0.001		
DBP-awakening(per 1 mmHg)	1.087(1.066–1.109)	<0.001		
SBP-bedtime(per 1 mmHg)	1.034(1.025–1.043)	<0.001		
DBP-bedtime(per 1 mmHg)	1.054(1.039–1.069)	<0.001		
24 h-SBP(per 1 mmHg)	1.067(1.054–1.081)	<0.001		
24 h-DBP(per 1 mmHg)	1.099(1.077–1.122)	<0.001	1.064(1.020–1.109)	<0.001
Clinic- SBP(per 1 mmHg)	1.043(1.033–1.052)	<0.001		
Clinic- DBP(per 1 mmHg)	1.039(1.025–1.053)	<0.001		
Reversed dipper SBP(1 = not reversed, 2 = reversed)	5.546(3.420–8.993)	<0.001	1.880(1.002–3.965)	0.036
Reversed dipper DBP(1 = not reversed, 2 = reversed)	4.082(2.572–6.478)	<0.001		

(Serum calcium is corrected by the following formula:[serum calcium] (mg/dL) = measured [serum calcium] (mg/dL)+(4.0−[serum albumin(mg/dl)]) × 0.8. Variables of univariate regression include age, sex, hemoglobin, serum calcium, phosphate and iPTH, all BP values from the clinic BP and ABPM, and reversed BP patterns, all variables with significant associations with eGFR were included in multivariate regression analysis. DBP: diastolic blood pressure; eGFR: estimated glomerular filtration rate; iPTH: intact parathyroid hormone, SBP: systolic blood pressure).

### 4. Proteinuria in these CKD Patients is not Related with a Reversed Dipper BP Pattern

Age, as well all BP levels from ABPM and the clinic were significantly related with proteinuria when univariate analysis was carried out. Multivariate logistic regression analyses revealed that bedtime SBP, clinical DBP and age were correlated with proteinuria ≥1 g/24 h. However, the reversed dipper BP pattern was not significantly related with proteinuria after two analyses (Table 3).

**Table 3:** Univariate and multivariate logistic regression analysis for proteinuria of 24 h (1 = proteinuria <1 g/24 h; 2 = proteinuria ≥1 g/24 h).

	Univariate regression analysis		Multivariate regression analysis	
	OR(95% CI)	P value	OR(95% CI)	P value
Age(per 1 y)	0.987(0.976–0.997)	0.012	0.979(0.968–0.991)	<0.001
Sex(1 = male, 2 = female)	1.006(0.712–1.420)	0.974		
eGFR<60 mL/min/1.73 m^2^	0.768(0.546–1.081)	0.130		
Hemoglobin(per 1 g/L)	1.000(0.994–1.005)	0.904		
Calcium(per 1 mg/dl)	0.988(0.957–1.109)	0.443		
Phosphate(per 1 mgl/dL)	1.076(0.769–1.507)	0.668		
iPTH(per 1 pg/ml)	1.000(0.999–1.000)	0.295		
Clinic- SBP(per 1 mmHg)	1.010(1.003–1.018)	0.005		
Clinic- DBP(per 1 mmHg)	1.025(1.012–1.038)	<0.001	1.025(1.006–1.045)	0.009
24 h-SBP(per 1 mmHg)	1.014(1.005–1.023)	0.002		
24 h-DBP(per 1 mmHg)	1.035(1.015–1.047)	<0.001		
SBP-awakening(per 1 mmHg)	1.015(1.005–1.024)	0.002		
DBP-awakening(per 1 mmHg)	1.028(1.011–1.024)	0.001		
SBP-bedtime(per 1 mmHg)	1.008(1.002–1.013)	0.009	1.008(1.002–1.015)	0.016
DBP-bedtime(per 1 mmHg)	1.011(1.001–1.021)	0.027		
Reversed dipper SBP(1 = not reversed, 2 = reversed)	1.171(0.785–1.147)	0.439		
Reversed dipper DBP(1 = not reversed, 2 = reversed)	0.806(0.544–1.194)	0.281		

(Serum calcium is corrected by the following formula:[serum calcium] (mg/dL) = measured [serum calcium] (mg/dL)+(4.0−[serum albumin(mg/dl)]) ×0.8. Variables of univariate regression include age, sex, eGFR(1 = eGFR≥60 mL/min/1.73 m^2^; 2 = eGFR<60 mL/min/1.73 m^2^), hemoglobin, serum calcium, phosphate and iPTH, all BP values from the clinic BP and ABPM, and reversed BP patterns, all variables with significant associations with eGFR were included in multivariate regression analysis. DBP: diastolic blood pressure; eGFR: estimated glomerular filtration rate; iPTH: intact parathyroid hormone, SBP: systolic blood pressure).

### 5. The Cardiovascular Index in these CKD Patients is Correlated with a Reversed Dipper BP Pattern

Age, sex, eGFR, levels of hemoglobin, serum calcium, phosphate and iPTH, all BP values from the clinic and ABPM, and reversed BP patterns were significantly correlated with the LVH by univariate analyses. Multivariate regression analyses revealed that female sex, lower eGFR, higher serum iPTH level, awakening SBP and reversed dipper DBP were independently correlated with LVH (Table 4). Data were shown in Table 4. Age, eGFR<60 mLl/min/1.73 m^2^, clinical BP, ABPM as well as the reversed dipper DBP and SBP were negatively correlated with the E/A ratio by univariate linear analyses, however, we only found age (β = –0.546, p<0.001) and DBP from AMBP (β = −0.375, p<0.001) to be independent factors for affecting the E/A ratio by multivariate linear regression analyses.

**Table 4:** Univariate and multivariate logistic regression analysis for left ventricular hypertrophy(1 = no LVH; 2 = LVH).

	Univariate regression analysis		Multivariate regression analysis	
	OR(95% CI)	P value	OR(95% CI)	P value
Age(per 1 y)	1.042(1.028–1.057)	<0.001		
Sex(1 = male, 2 = female)	1.667(1.086–2.558)	<0.001	3.502(1.911–6.419)	<0.001
eGFR<60 ml/min/1.73 m^2^	9.227(5.136–16.579)	<0.001	2.709(1.309–5.607)	0.007
Hemoglobin(per 1 g/L)	0.967(0.959–0.975)	<0.001		
Calcium(per 1 mg/dl)	0.261(0.107–0.663)	0.003		
Phosphate(per 1 mgl/dL)	3.168(2.069–4.850)	<0.001		
iPTH(per 1 pg/ml)	1.004(1.002–1.005)	<0.001	1.002(1.001–1.003)	0.002
Clinic- SBP(per 1 mmHg)	1.041(1.031–1.052)	<0.001		
Clinic- DBP(per 1 mmHg)	1.031(1.015–1.047)	<0.001		
24 h-SBP(per 1 mmHg)	1.065(1.049–1.081)	<0.001		
24 h-DBP(per 1 mmHg)	1.068(1.045–1.091)	<0.001		
SBP-awakening(per 1 mmHg)	1.068(1.052–1.085)	<0.001	1.061(1.041–1.081)	<0.001
DBP-awakening(per 1 mmHg)	1.058(1.036–1.080)	<0.001		
SBP-bedtime(per 1 mmHg)	1.045(1.033–1.057)	<0.001		
DBP-bedtime(per 1 mmHg)	1.063(1.043–1.082)	<0.001		
Reversed dipper SBP(1 = not reversed, 2 = reversed)	2.921(1.823–4.681)	<0.001		
Reversed dipper DBP(1 = not reversed, 2 = reversed)	4.110(2.578–6.552)	<0.001	2.375(1.286–4.384)	0.006

(Serum calcium is corrected by the following formula:[serum calcium] (mg/dL) = measured [serum calcium] (mg/dL)+(4.0−[serum albumin(mg/dl)]) ×0.8. Variables of univariate regression include age, sex, eGFR(1 = eGFR≥60 mL/min/1.73 m^2^; 2 = eGFR<60 mL/min/1.73 m^2^), hemoglobin, serum calcium, phosphate and iPTH, all BP values from the clinic BP and ABPM, and reversed BP patterns, all variables with significant associations with eGFR were included in multivariate regression analysis. DBP: diastolic blood pressure; eGFR: estimated glomerular filtration rate; LVH: left ventricular hypertrophy; iPTH: intact parathyroid hormone, SBP: systolic blood pressure).

### 6. Factors Affecting the Reversed Dipper BP Pattern in CKD Patients

Univariate regression analyses revealed older age, female sex, higher BP from ABPM and the clinic, eGFR<60 mL/min/1.73 m^2^, lower hemoglobin level, and higher serum levels of calcium, phosphate and iPTH to be significantly correlated with a reversed BP pattern. However, we found only older age, higher 24-h SBP from ABPM, lower hemoglobin and higher serum phosphate levels to be independently correlated with the reversed BP pattern after multivariate regression analyses (Table 5).

**Table 5:** Univariate and multivariate logistic regression analysis for reversed dipper blood pressure pattern (1 (1 = not reversed dipper, 2 = reversed dipper)).

	Univariate regression analysis		Multivariate regression analysis	
	OR(95% CI)	P value	OR(95% CI)	P value
Age((per 1 y)	1.040(1.027–1.054)	<0.001	1.021(1.005–1.037)	0.011
Sex(1 = male, 2 = female)	1.582(1.061–2.358)	0.024		
eGFR<60 mL/min/1.73 m^2^	4.082(2.572–6.478)	<0.001		
Hemoglobin(per 1 g/L)	0.971(0.963–0.978)	<0.001	0.984(0.974–0.994)	0.002
Calcium(per 1 mg/dl)	0.296(0.129–0.677)	0.004		
Phosphate(per 1 md/dlL)	3.121(2.114–4.607)	<0.001	1.666(1.012–2.742)	0.045
iPTH(per 1 pg/ml)	1.002(1.001–1.003)	<0.001		
Clinic- SBP(per 1 mmHg)	1.019(1.011–1.028)	<0.001		
Clinic- DBP(per 1 mmHg)	1.006(0.992–1.020)	0.392		
24 h-SBP(per 1 mmHg)	1.036(1.024–1.047)	<0.001	1.019(1.015–1.033)	0.007
24 h-DBP(per 1 mmHg)	1.029(1.011–1.048)	<0.001		

(Serum calcium is corrected by the following formula:[serum calcium] (mg/dL) = measured [serum calcium] (mg/dL)+(4.0−[serum albumin(mg/dl)]) ×0.8. Variables of univariate regression include age, sex, eGFR(1 = eGFR≥60 mL/min/1.73 m^2^; 2 = eGFR<60 mLl/min/1.73 m^2^), levels of hemoglobin, serum calcium, phosphate and iPTH, Clinic- SBP and DBP and 24 h-SBP and DBP. All variables with significant associations with blood pressure pattern were included in multivariate regression analysis. DBP: diastolic blood pressure; eGFR: estimated glomerular filtration rate; iPTH: intact parathyroid hormone, SBP: systolic blood pressure).

## Discussion

We initially reported the prevalence of reversed dipper BP pattern in Chinese CKD patients. These patients had worse renal function, more severe anemia and disturbance in calcium phosphate metabolism, and worse cardiovascular damage compared with subjects with a dipper BP pattern. These findings were similar to those of previous reports [16]–[18]. Previous studies have focused on mixed groups comprising individuals with non-dipper and a reversed dipper BP patterns, and data solely on patients with a reversed BP pattern in Chinese CKD patients are lacking. We found that patients with reversed dipper BP pattern had worse renal function and cardiovascular damages even compared with patients with non-dipper BP pattern. We also found that the rate of decline of nocturnal BP correlated with the eGFR, LVMI and E/A ratio. This finding suggested that the increase in nocturnal BP (and also the reversed dipper BP status) correlated with these three parameters. This is consistent with our findings that patients with a reversed dipper BP pattern have the worst kidney damage and cardiovascular damage among CKD patients. We also found that a reversed dipper BP pattern was independently correlated with kidney damage and LVH in CKD patients. All these data suggested that a reversed dipper BP pattern was correlated with target-organ damage in CKD patients, however, we need more studies to get cause-effect relationship between BP pattern and target organ damages. It might be suggested that ABPM should be use for CKD patients so that more information on BP can be obtained and individuals with a reversed dipper BP pattern can be identified. We should pay special attention to these patients and take measures such as antihypertensive “chronotherapy”. Such treatment might efficiently reduce nocturnal BP and improve the circadian pattern in CKD without additional cost [19], [20].

A reversed dipper BP means that nocturnal BP is higher than daytime BP, which is opposite to the physiological rhythm of blood pressure. Nocturnal BP represents the minimal BP that is needed for adequate organ perfusion in healthy subjects [21]. Maintaining a high BP at night, however, overloads the cardiovascular system, with a consequent negative impact on the heart and vascular structures. Likewise, in the kidney, the resting period is when the afferent arteriolar tone is lower, thereby allowing for more direct transmission of the systemic BP to the glomerulus. Consequently, a high BP at night impacts the heart, vasculature, and kidney, boosting damage and increasing risk of developing clinical events [22]. In the present study, it was not surprising to find that patients with reversed dipper BP pattern had the most severe renal and cardiovascular injuries among CKD patients, and that the reversed dipper BP pattern was closely related to target-organ damage. Therefore, lowering nocturnal BP might help to reduce cardiovascular and renal risk for patients with a reversed dipper BP.

The mechanism of reversed or non-dipper BP patterns remains unknown. It could be a consequence of autonomic dysfunction, relative nocturnal volume overload, and abnormal handling of sodium. One study has shown that hypertension (especially elevated night-time BP) is associated with increased night-time sodium excretion, and that the correlation between night-time sodium excretion and night-time BP is stronger than the correlation between daytime sodium excretion and daytime BP [23]. One explanation for this phenomenon is that a diminished renal capacity to excrete sodium causes nocturnal BP elevation, which enhances pressure natriuresis in compensation for impaired daytime natriuresis. Findings have demonstrated that patients with renal dysfunction require a longer time until BP falls during the night [24]. The present study suggests that subjects with a reversed dipper BP pattern have a higher ratio of bedtime/awakening time of urinary sodium excretion compared with those with a non-dipper BP pattern. Our previous studies suggested that a higher ratio of bedtime/awakening urine sodium excretion could be found in patients with a non-dipper BP pattern, which can be lowered along with the changes in the non-dipper BP pattern [25]. This finding suggests that abnormal handling of sodium by injured kidney might be a key factor for a non-dipper BP pattern in CKD. We also found that the main factors contributing to a reversed dipper BP pattern were SBP, hemoglobin level and phosphate level. Higher BP levels might indicate that self-regulating mechanisms are impaired, so it was not surprising to find that subjects with a reverse dipper BP pattern had the highest BP levels among CKD patients. Levels of anemia and disturbances in calcium-phosphate metabolism in CKD patients are important clinical markers to define the severity of CKD and are closely related to renal loss. It is therefore unsurprising that we found the most serious anemia and calcium-phosphate metabolism disturbances in patients with reversed dipper BP pattern. We also reported that serum phosphate levels were independently correlated with the reversed BP pattern after multivariate regression analyses. Kanbay et al reported a similar result in that non-dipper patients had higher levels of phosphate, Ca×P product and PTH compared with dipper patients, and independent predictors for non-dipper hypertension were levels of PTH and phosphate [26]. There are also some differences between our results and data from Kanbay, which may be related to different areas (China *vs* Turkey), size (540 *vs* 190) and target (reversed dipper BP pattern vs non-dipper patten). However these two studies undoubtedly indicated that serum phosphate levels might affect the rate of decline of nocturnal BP. More studies are needed to explore the real mechanism.

The present study had several strengths. We are the first to report the prevalence of reverse BP patterns in Chinese CKD patients and their close relationship with target-organ damage. Secondly, the sample size was large considering that CKD patients were our selected cohort. Finally, we reported ABPM results, renal damage and cardiovascular damage in Chinese CKD patients. However, some limitations should be considered when evaluating our results. Firstly, this was an observational study and we did not provide a standard clinical trial to change the reversed dipper blood pressure pattern. Secondly, long-term follow-up data were not provided. Finally, we can not get the cause-effect relationship between BP pattern and target organ damages.

### Conclusions

A reversed dipper BP pattern is closely correlated with severe renal damage and cardiovascular injuries in Chinese CKD patients, and special attention should be given to subjects with a reversed dipper BP pattern. More clinical trials are needed to confirm the role of a reverse dipper pattern and high nocturnal BP on CKD progression.
